# Texture indices of 4′-[methyl-^11^C]-thiothymidine uptake predict p16 status in patients with newly diagnosed oropharyngeal squamous cell carcinoma: comparison with ^18^F-FDG uptake

**DOI:** 10.1186/s41824-020-00090-y

**Published:** 2020-11-02

**Authors:** Ayumi Ihara-Nishishita, Takashi Norikane, Katsuya Mitamura, Yuka Yamamoto, Kengo Fujimoto, Yasukage Takami, Emi Ibuki, Nobuyuki Kudomi, Hiroshi Hoshikawa, Jun Toyohara, Yoshihiro Nishiyama

**Affiliations:** 1 Department of Radiology, Faculty of Medicine, Kagawa University, 1750-1 Ikenobe, Miki-cho, Kita-gun, Kagawa 761-0793 Japan; 2 Department of Diagnostic Pathology, Faculty of Medicine, Kagawa University, Kagawa, Japan; 3 Department of Medical Physics, Faculty of Medicine, Kagawa University, Kagawa, Japan; 4 Department of Otolaryngology, Faculty of Medicine, Kagawa University, Kagawa, Japan; 5 Research Team for Neuroimaging, Tokyo Metropolitan Institute of Gerontology, Tokyo, Japan

**Keywords:** ^11^C-4DST, ^18^F-FDG, PET, Oropharyngeal squamous cell carcinoma, Texture

## Abstract

**Background:**

In oropharyngeal squamous cell carcinoma (OPSCC), human papillomavirus (HPV)/p16 status is important as a prognostic biomarker.

**Purpose:**

We evaluated the relationship between 4′-[methyl-^11^C]-thiothymidine (^11^C-4DST) and ^18^F-FDG PET texture indices and p16 status in patients with newly diagnosed OPSCC.

**Methods:**

We retrospectively reviewed the collected data of 256 consecutive, previously untreated patients with primary head and neck tumors enrolled between November 2011 and October 2019. Complete data on both ^11^C-4DST and ^18^F-FDG PET/CT studies before therapy, patients with OPSCC, and p16 status were available for 34 patients. Six of them were excluded because they did not exhibit sufficient ^11^C-4DST and/or ^18^F-FDG tumor uptake to perform textural analysis. Finally, 28 patients with newly diagnosed OPSCC were investigated. The maximum standardized uptake value (SUVmax) and 6 texture indices (homogeneity, entropy, short-run emphasis, long-run emphasis, low gray-level zone emphasis, and high gray-level zone emphasis) were derived from PET images. The presence of p16 expression in tumor specimens was examined by immunohistochemistry and compared with the PET parameters.

**Results:**

Using ^11^C-4DST, the expression of p16 was associated with a higher homogeneity (*P* = 0.012), lower short-run emphasis (*P* = 0.005), higher long-run emphasis (*P* = 0.009), and lower high-gray-level-zone emphasis (*P* = 0.042) values. There was no significant difference between ^18^F-FDG PET parameters and p16 status.

**Conclusion:**

Texture indices of the primary tumor on ^11^C-4DST PET, but not ^18^F-FDG PET, may be of value in predicting the condition’s p16 status in patients with newly diagnosed OPSCC.

## Introduction

Identifying the imaging biomarkers of tumors is very important because this information can provide useful targets for treatment without the requirement for tissue sampling (Schillaci and Urbano 2017). Study in the sphere of imaging genomics (also referred to as radiogenomics) has shown the potential in getting tumor genotypes and phenotypes (Schillaci and Urbano 2017). Human papillomavirus (HPV)-related oropharyngeal squamous cell carcinoma (OPSCC) has been built up as a biologically distinct from HPV-negative OPSCC (Ang et al. 2010). HPV-related OPSCC, usually identified by p16 as a surrogate marker, more responsive to therapy and with a better prognosis (Ang et al. 2010; Posner et al. 2011). Despite the extensive research already devoted to the biological and clinical behavior of HPV-related OPSCC, its imaging characteristics have been paid relatively little attention.

Positron emission tomography (PET) with 2-deoxy-2-^18^F-fluoro-D-glucose (^18^F-FDG) is a useful functional tool for the diagnosis and surveillance of head and neck squamous cell carcinoma (HNSCC) (Bonomo et al. 2018). While ^18^F-FDG directly reflects the glucose metabolism, Toyohara et al. developed 4′-[methyl-^11^C]-thiothymidine (^11^C-4DST) for cell proliferation imaging that is resistant to degradation by thymidine phosphorylase and is incorporated into DNA (Toyohara et al. 2006, 2008, 2011). ^11^C-4DST PET was found by Hoshikawa et al. to provide important prognostic value in patients with HNSCC (Hoshikawa et al. 2017). The most commonly used PET semiquantitative parameter is the maximum standardized uptake value (SUVmax). Joo et al. documented an association between higher ^18^F-FDG SUVmax for primary tumor and HPV-negative OPSCC (Joo et al. 2014). Another study, though, detected no significant relationship between ^18^F-FDG SUV parameters for primary tumor and HPV status in patients with OPSCC or oral cavity squamous cell carcinoma (Kendi et al. 2015). Recently, increasing attention is being turned to measurements of tumor heterogeneity based on texture analysis. Chan et al. found ^18^F-FDG PET heterogeneity to be prognostically superior to traditional SUV parameters in patients with pharyngeal cancer (Chan et al. 2017). Several investigators have focused on the textural features of ^18^F-FDG PET in patients with HNSCC, but the number of such studies remains very small (Chan et al. 2017; Chen et al. 2017; Cheng et al. 2015; Wang et al. 2016; Fujima et al. 2018).

As far as we could determine, no published report has focused on the relationship between PET textural parameters and HPV/p16 status in patients with OPSCC. With this in mind, we evaluated the relationship between ^11^C-4DST and ^18^F-FDG PET texture indices and p16 status in patients with newly diagnosed OPSCC.

## Materials and methods

### Patients

We conducted a retrospective analysis of prospectively collected data. The study cohort consisted of 256 consecutive, previously untreated patients with primary head and neck tumors enrolled between November 2011 and October 2019. Complete data on both ^11^C-4DST and ^18^F-FDG PET/CT studies before therapy, patients with OPSCC, and p16 status were available for 34 patients from July 2013 to October 2019. Six of them were excluded because they did not exhibit sufficient ^11^C-4DST and/or ^18^F-FDG tumor uptake to perform textural analysis. Finally, 28 patients (25 males, 3 females; mean age, 66.5 years; age range, 52-87 years) were enrolled in the study. Their clinical data are summarized in Table 1. The study protocol was approved by our institutional ethics review committee. The requirement for informed consent was waived due to its retrospective nature.

**Table 1:** Clinical characteristics of the 28 patients with newly diagnosed oropharyngeal squamous cell carcinoma

Characteristic	Value
Age (years)	
Mean	66.5
Range	52-87
Sex (*n*)	
Male	25
Female	3
Primary site (*n*)	
Tonsil	21
Soft palate	4
Base of tongue	2
Posterior pharyngeal wall	1
T stage (*n*)	
T1	1
T2	13
T3	3
T4	11
Smoking history (*n*)	
Yes	24
No	4
Alcohol history (*n*)	
Yes	22
No	6
p16 status (*n*)	
Positive	13
Negative	15

### Radiotracer synthesis and PET/CT imaging

^11^C-4DST and ^18^F-FDG were produced using an automated synthesis system with HM-18 cyclotron (QUPID; Sumitomo Heavy Industries Ltd., Tokyo, Japan). The ^11^C-4DST was synthesized using the method mentioned by Toyohara et al. (2011).

All acquisitions were performed using a Biograph mCT 64-slice PET/CT scanner (Siemens Medical Solutions USA Inc., Knoxville, TN, USA), which has an axial field of view of 21.6 cm. The mean time interval between ^11^C-4DST and ^18^F-FDG PET/CT scans was 6 days (range 0–24 days).

Patients were instructed to fast for at least 5 h before ^18^F-FDG administration. A normal glucose level in the peripheral blood was confirmed before the injection. PET emission scanning (2 min per bed position) was performed 15 min after intravenous injection of ^11^C-4DST (7.4 MBq/kg) and 90 min after intravenous injection of ^18^F-FDG (3.7 MBq/kg) from the midcranium to the proximal thighs, and co-registered with an unenhanced CT of the same region (Quality Reference mAs: 100 mAs [using CARE Dose4D]; reconstructed slice thickness: 5 mm). The PET data were reconstructed with a baseline ordered-subset expectation maximization algorithm, incorporating correction with point-spread function and time-of-flight model (2 iterations, 21 subsets). A Gaussian filter with a full-width at half-maximum of 5 mm was used as a post-smoothing filter.

### Image analyses

The LIFEx software was used to extract the texture indices of PET images from the volume of interest (VOI) of the primary tumor (Nioche et al. 2018). The patients’ PET images in DICOM format were imported into this software. A board-certified nuclear medicine physician used the 40% threshold of SUVmax to semi-automatically set the primary tumor. If non-tumoral areas of activity were incorrectly included within the VOI, adjustments were performed by the operator.

The SUVmax was calculated using the following formula: SUV = *c*_dc_/(*d*_i_/*w*), where *c*_dc_ is the decay-corrected tracer tissue concentration (Bq/g); *d*_i_, the injected dose (Bq); and *w*, the patient’s body weight (g).

Six texture indices (homogeneity, entropy, short-run emphasis (SRE), long-run emphasis (LRE), low gray-level zone emphasis (LGZE), and high gray-level zone emphasis (HGZE)) were calculated according to a report by Orlhac et al. (2014, 2017).

### Immunohistochemistry

Paraffin-embedded samples of the primary tumor obtained by surgical resection (*n* = 6) and biopsy (*n* = 22) were immunostained for p16. Staining was performed using the labeled streptavidin biotinylated antibody method with an autostaining system (Ventana Benchmark System, Ventana Medical Systems, Tucson, AZ, USA) according to the manufacturer’s protocol. Mouse monoclonal antibody against p16/INK4a (Ventana Medical Systems, Tucson, AZ, USA) was used as the primary antibody. Staining of p16 was considered positive when strong nuclear and cytoplasmic staining was present in 75% or more of the tumor cells (Lydiatt et al. 2017).

### Statistical analyses

All statistical analyses were performed using a software package (SPSS Statistics, version 26; IBM). The ^11^C-4DST and ^18^F-FDG SUVmax values were compared using the paired *t* test. The differences between PET parameters and p16 status were compared using the Mann-Whitney *U* test, and were considered statistically significant at *P* values less than 0.05.

## Results

Primary tumors were detected in all patients on both ^11^C-4DST and ^18^F-FDG PET images. The mean (± SD) SUVmax for ^11^C-4DST (8.49 ± 2.25) was significantly lower than that for ^18^F-FDG (16.78 ± 8.19) (*P* < 0.001).

**Fig. 1:**
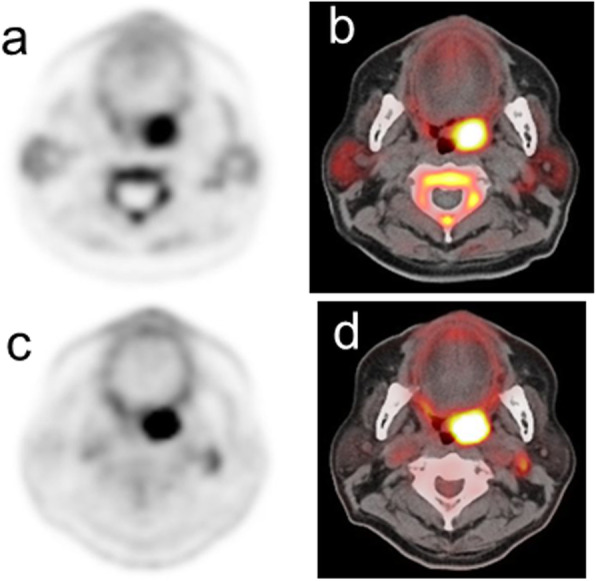
PET/CT images from a 67-year-old male diagnosed with p16-positive oropharyngeal squamous cell carcinoma in the left palatine tonsil. Transverse ^11^C-4DST PET image (**a**) and fused PET/CT image (**b**) show an increased uptake in the primary tumor (SUVmax = 7.41, homogeneity = 0.344, entropy = 2.130, SRE = 0.948, LRE = 1.220, LGZE = 0.00676, and HGZE = 199.90). Transverse ^18^F-FDG PET image (**c**) and fused PET/CT image (**d**) also show an increased uptake in the primary tumor (SUVmax = 14.42, homogeneity = 0.206, entropy = 2.230, SRE = 0.979, LRE = 1.080, LGZE = 0.00180, and HGZE = 867.60)

**Fig. 2:**
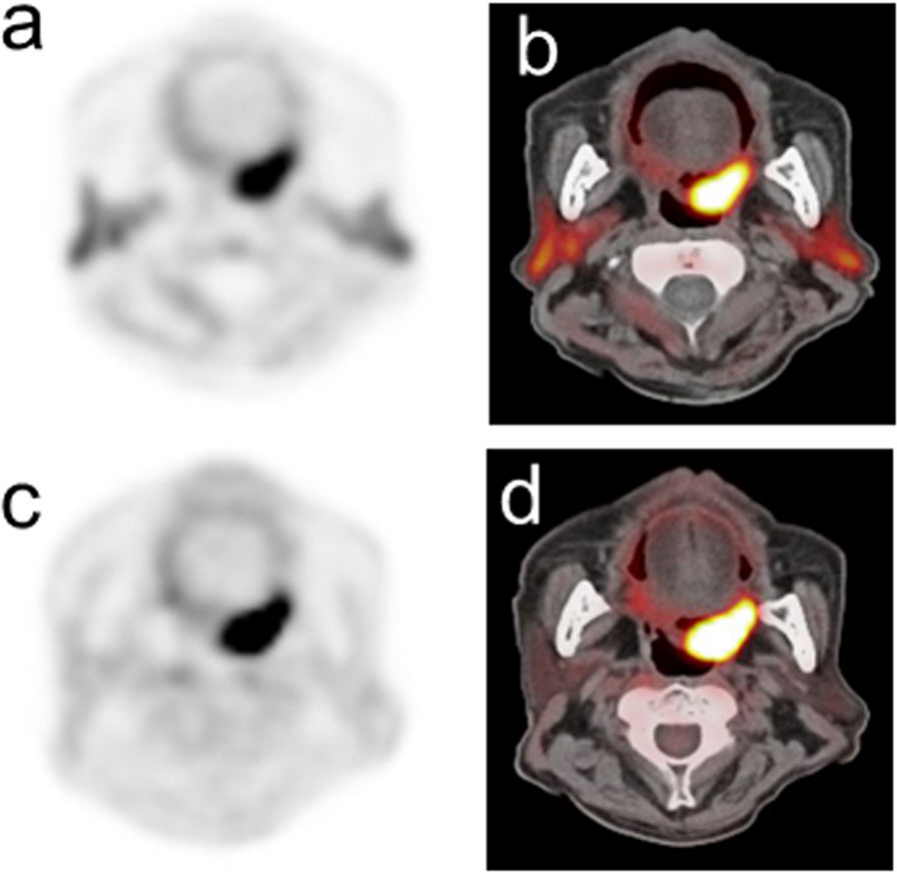
PET/CT images from a 66-year-old male diagnosed with p16-negative oropharyngeal squamous cell carcinoma in the soft palate. Transverse ^11^C-4DST PET image (**a**) and fused PET/CT image (**b**) show an increased uptake in the primary tumor (SUVmax = 8.00, homogeneity = 0.295, entropy = 2.050, SRE = 0.965, LRE = 1.140, LGZE = 0.00514, and HGZE = 260.00). Transverse ^18^F-FDG PET image (**c**) and fused PET/CT image (**d**) also show an increased uptake in the primary tumor (SUVmax = 14.57, homogeneity = 0.194, entropy = 1.970, SRE = 0.986, LRE = 1.060, LGZE = 0.00168, and HGZE = 843.90)

Table 2 summarizes the PET semiquantitative parameters in relation to p16 status. There was no significant difference in SUVmax values between either ^11^C-4DST or ^18^F-FDG PET and p16 status. Using ^11^C-4DST PET, the expression of p16 was associated with a higher homogeneity (*P* = 0.012), lower SRE (*P* = 0.005), higher LRE (*P* = 0.009), and lower HGZE (*P* = 0.042) values. None of the 6 texture indices using ^18^F-FDG PET showed significant differences between p16-positive and p16-negative tumors. Typical PET/CT images from p16 positive and p16 negative patient are shown in Figs. 1 and 2, respectively.

**Table 2:** Primary tumor semiquantitative PET parameters and p16 status data for the 28 patients with newly diagnosed oropharyngeal squamous cell carcinoma

PET parameter	p16 positive (*n* = 13)		p16 negative (*n* = 15)		*P* value
	Mean	SD	Mean	SD	
^11^C-4DST					
SUVmax	7.79	1.48	9.10	2.64	0.066
Homogeneity	0.360	0.360	0.330	0.090	0.012
Entropy	2.078	0.177	2.058	0.273	0.857
SRE	0.942	0.014	0.950	0.037	0.005
LRE	1.272	0.077	1.241	0.240	0.009
LGZE	0.00634	0.00210	0.00499	0.00265	0.053
HGZE	241.08	101.89	346.99	172.82	0.042
^18^F-FDG					
SUVmax	15.29	6.09	18.08	9.68	0.684
Homogeneity	0.253	0.062	0.305	0.177	0.822
Entropy	2.147	0.261	1.920	0.538	0.255
SRE	0.963	0.023	0.930	0.108	0.822
LRE	1.183	0.180	1.665	1.419	0.857
LGZE	0.00263	0.00177	0.00193	0.00131	0.619
HGZE	970.54	640.41	1134.29	799.85	0.651

*SUVmax* maximum standardized uptake value, *SRE* short-run emphasis, *LRE* long-run emphasis, *LGZE* low gray-level zone emphasis, *HGZE* high gray-level zone emphasis

## Discussion

HPV/p16 status has come to be recognized as an important risk factor and prognostic biomarker for HNSCC, especially OPSCC (Ang et al. 2010; Posner et al. 2011). We believe the present study to be the first to focus on the relation between ^11^C-4DST heterogeneity and the expression of p16, in patients with newly diagnosed OPSCC, as compared with ^18^F-FDG. Our findings suggest that four texture indices of the primary OPSCC on ^11^C-4DST PET may be of value in recognizing the condition’s p16 status.

^11^C-4DST closely mimics the thymidine metabolism. ^11^C-4DST is incorporated into the DNA via salvage pathway in rapidly proliferating tissues. The phosphorylating enzyme, thymidine kinase 1 (TK1) is the rate-limiting step of salvage pathway. Therefore, the DNA incorporation rate of ^11^C-4DST in tumor cells is affected by the activity of TK1. The causal role of HPV in OPSCC depends on the activity of the viral oncoproteins E6 and E7 (Taberna et al. 2017). The E6 binds to the tumor suppressor protein p53, which results in p53 degradation. The retinoblastoma gene product pRB is a target of the E7 oncoprotein. Functional inactivation of p53 and pRB leads to compensatory overexpression of p16, the inhibitor of cyclin-dependent kinases, in a negative feedback loop (Yokota 2014). Interestingly, the expression of TK1 gene is regulated by the E2F transcription factor and positioned in the lowest stream of the pRB pathway, a common pathway of cell proliferation regulation signals (Bartek et al. 1996; Ewen 1994; Sherr 1995; Sherr and Roberts 1995; Weinberg 1995). p16 over expression in OPSCC reflects the inactivation of pRB. Inactivation of pRB releases E2F transcription factor and increases the expressions of TK1. As a result, p16 over expression and tumor uptake pattern of ^11^C-4DST might have some indirect interactions. Therefore, it is reasonable to consider some relationship between texture indices of ^11^C-4DST PET and p16 status was detected. However, the biological meanings of this findings are not clear.

Several researches have investigated the association between ^18^F-FDG PET findings and tumor HPV/p16 status in patients with HNSCC (Joo et al. 2014; Kendi et al. 2015; Chen et al. 2017; Tahari et al. 2014; Surov et al. 2019; Schouten et al. 2016; Huang et al. 2015). Some noted ^18^F-FDG PET SUV parameters to be significantly higher in HPV-negative as compared with HPV-positive primary OPSCC (Joo et al. 2014; Tahari et al. 2014; Surov et al. 2019; Schouten et al. 2016). However, such results have not always been consistent concerning the correlation between ^18^F-FDG PET parameters and HPV/p16 status (Kendi et al. 2015; Chen et al. 2017; Huang et al. 2015). Chen et al. demonstrated that the expression of p16 was not related to the textural features on ^18^F-FDG PET in patients with pharyngeal cancer (Chen et al. 2017). Huang and colleagues did not observe a significant relationship either between ^18^F-FDG SUV parameters including volumetric factors and p16 status in patients with OPSCC (Huang et al. 2015). In the present study, we similarly did not identify significant correlations between SUVmax values and textural parameters on ^18^F-FDG PET and expression of p16 status. This discrepancy in the significance of semiquantitative ^18^F-FDG PET parameters may be attributable to differences in patient populations and in imaging protocols as well as image analyses or as yet other unidentified factors in the respective studies.

To date, there are no reports comparing ^11^C-4DST parameters and HPV/p16 status in patients with OPSCC. In the present study, the expression of p16 associated with higher homogeneity, lower SRE, higher LRE, and lower HGZE values on ^11^C-4DST PET, although this association was not present on ^11^C-4DST SUVmax. The prognostic information of ^18^F-FDG PET textural indices in patients with pharyngeal cancer has been studied (Chan et al. 2017; Chen et al. 2017; Cheng et al. 2013, 2015; Wang et al. 2016; Fujima et al. 2018). Chan et al. proved that heterogeneity on ^18^F-FDG PET was prognostically superior to traditional SUV parameters in such patients (Chan et al. 2017). According to Fujima et al., higher ^18^F-FDG homogeneity was an independent predictor of prognosis in patients with pharyngeal cancer, although this association was not present in the case of ^18^F-FDG SUV parameters (Fujima et al. 2018). Chen et al. also demonstrated that ^18^F-FDG heterogeneity indices were more informative than classical SUV indices in the prediction of patient prognosis in pharyngeal cancer (Chen et al. 2017). Taken together, the textural indices compared to classical SUV parameters might have a role in determining the prognosis in patients with pharyngeal cancer. Such findings are attributable to the inability of classical PET parameters such as SUV to delineate tumor heterogeneity, which has been explained by a number of underlying factors such as cellular proliferation, cellularity, angiogenesis, necrosis, and vascularization (Huang et al. 2015). However, the exact biologic correlates of these PET heterogeneity parameters remain to be determined. Further studies will be needed to investigate the relation between PET textural parameters and tumor biology in patients with a variety of tumor types.

Limitations of the present study include its small sample size and retrospective design. The histopathological samples represent only a small portion of the tumors, whereas PET was analyzed as a whole tumor measurement. Only p16 was used as a surrogate marker for HPV infections because HPV DNA testing was not available. We did not analyze Ki-67 status at histopathological samples. Tsuchida et al. reported that Ki-67 positivity was commonly observed for both HPV-positive and HPV-negative tumors of OPSCC (Tsuchida et al. 2017). Some studies have proposed that HPV-related tumors have high radiosensitivity, which may explain the favorable prognosis of patients with HPV-positive OPSCC (Wang et al. 2020). The increased radiosensitivity of HPV-positive cells may be attributed to the improvement of the intracellular hypoxic environment (Lassen et al. 2010; Wang et al. 2020). We did not compare the PET parameters studied and the presence of hypoxia at histopathological samples. Additional large prospective studies will be needed to verify and expand the present results.

A more important indication is the possibility that cell proliferation imaging could be used for the assessment of early response to therapy. In addition, p16 is one of the relevant prognostic markers in patients with OPSCC. The role of ^11^C-4DST PET in therapy monitoring has not been evaluated thus far. Chan et al. found the combination of ^18^F-FDG PET heterogeneity parameters and dynamic contrast-enhanced MRI parameters to be beneficial in the prediction of patient prognosis in pharyngeal cancer (Chan et al. 2017). Advances in hardware such as simultaneous PET/MRI will help to further facilitate imaging research on analysis of tumor heterogeneity. Few tumor heterogeneity studies have yet been undertaken using newer radiopharmaceuticals other than ^18^F-FDG. Further studies will be needed to evaluate texture parameters using different imaging tools and different radiopharmaceuticals to clarify their potential clinical information.

## Conclusion

Regarding the results of this preliminary study, the paucity of our data in our small patient population precludes any definite conclusion. Despite this, it was possible to document associations between the expression of p16 with higher homogeneity, lower SRE, higher LRE, and lower HGZE values on ^11^C-4DST PET in patients with newly diagnosed OPSCC, while notably, these associations were not present on ^18^F-FDG PET.

## Data Availability

All datasets used during the current study are available from the corresponding author on reasonable request.

## Abbreviations

OPSCC: Oropharyngeal squamous cell carcinoma
HPV: Human papillomavirus
PET: Positron emission tomography
^18^F-FDG: 2-deoxy-2-^18^F-fluoro-D-glucose
HNSCC: Head and neck squamous cell carcinoma
^11^C-4DST: 4′-[methyl-^11^C]-thiothymidine
SUVmax: Maximum standardized uptake value
VOI: Volume of interest
SRE: Short-run emphasis
LRE: Long-run emphasis
LGZE: Low gray-level zone emphasis
HGZE: High gray-level zone emphasis
